# Author Correction: Preserved wake-dependent cortical excitability dynamics predict cognitive fitness beyond age-related brain alterations

**DOI:** 10.1038/s42003-021-01995-5

**Published:** 2021-04-08

**Authors:** Maxime Van Egroo, Justinas Narbutas, Daphne Chylinski, Pamela Villar González, Pouya Ghaemmaghami, Vincenzo Muto, Christina Schmidt, Giulia Gaggioni, Gabriel Besson, Xavier Pépin, Elif Tezel, Davide Marzoli, Caroline Le Goff, Etienne Cavalier, André Luxen, Eric Salmon, Pierre Maquet, Mohamed Ali Bahri, Christophe Phillips, Christine Bastin, Fabienne Collette, Gilles Vandewalle

**Affiliations:** 1 GIGA-Cyclotron Research Centre-In Vivo Imaging, University of Liège, Liège, Belgium; 2 Psychology and Cognitive Neuroscience Research Unit, University of Liège, Liège, Belgium; 3 Department of Clinical Chemistry, University Hospital of Liège, Liège, Belgium; 4 Department of Neurology, University Hospital of Liège, Liège, Belgium; 5 GIGA-In Silico Medicine, University of Liège, Liège, Belgium

**Keywords:** Wakefulness, Cognitive ageing

Correction to: *Communications Biology* 10.1038/s42003-019-0693-y, published online 3 December 2019.

In the original version of the article, authors found that there had been an overestimation of overnight slow wave energy for a subset of individuals for which the data are presented in Fig. 3

In the original version of the article, Fig. 3 appeared as:

All three panels have been replaced so in the new version of the article Fig. 3 appears as:

As a result, some text in the manuscript has also been replaced.

- In the “Methods” section on page 8, under the heading of “Sleep assessment and spectral power analysis,” the text:
  “The night was divided into 30-min periods, from sleep onset until lights on. For each 30-min period, SWE was computed as the sum of generated power in the delta band, both for the lower range (0.75–1 Hz) and higher range (1.25–4 Hz), during all the NREM 2 and NREM 3 epochs of the given period, after adjusting for the number of NREM 2 and 3 epochs to account for artefacted data.”
  has been replaced with:
  “The night was divided into 30-min periods, from sleep onset until lights on. For each 30-min period, SWE was computed as the sum of generated power in the delta band, both for the lower range (0.5–1 Hz) and higher range (1.25–4 Hz), during all the NREM 2 and NREM 3 epochs of the given period, after adjusting for the number of NREM 2 and 3 epochs to account for artefacted data.”
- In the “Results” section on page 3, under the heading “CEP and slow waves generation” the text:
  “We then confronted the validity of frontal CEP as a measure of sleep–wake regulation by testing its association with slow waves generation during sleep^26^. We found that frontal CEP was significantly related to slow wave energy (SWE), a cumulative measure of slow waves generated during NREM sleep, both in the slower range (0.75–1 Hz, *F*_1,55_ = 5.35, *p* = 0.02, *R*²_β*_ = 0.09, Fig. 3a) and in the higher range (1.25–4 Hz, *F*_1,55_ = 5.47, *p* = 0.02, *R*²_β*_ = 0.09, Fig. 3b), such that young-like frontal CEP was associated with increased, and presumably preserved SWE. We further found that SWE in the lower 0.75–1 Hz range was expectedly associated with GM volume (*F*_1,53_ = 7.90, *p* = 0.007, *R*²_β*_ = 0.13), as well as with whole-brain Aβ burden (*F*_1,53_ = 5.15, *p* = 0.03, *R*²_β*_ = 0.09, Fig. 3c), confirming previous reports^11,27^, but was not linked to whole-brain tau burden (*F*_1,53_ = 1.26, *p* = 0.27). In contrast, CEP was not associated with any of the brain structural integrity measures (GM: *F*_1,53_ = 0.18, *p* = 0.67; Aβ: *F*_1,53_ = 0.16, *p* = 0.69; Tau: *F*_1,53_ = 0.48, *p* = 0.49). Frontal CEP relates therefore to a gold standard measure of sleep homeostasis known to decline in aging^13,15^ and to be associated with Aβ burden^11^, but is not significantly linked to the hallmarks of AD neuropathology.”
  has been replaced with:
  “We then confronted the validity of frontal CEP as a measure of sleep–wake regulation by testing its association with slow waves generation during sleep^26^. We found that frontal CEP was significantly related to slow wave energy (SWE), a cumulative measure of slow waves generated during NREM sleep, both in the slower range (0.5–1 Hz, *F*_1,55_ = 4.72, *p* = 0.03, *R*²_β*_ = 0.08, Fig. 3a) and in the higher range (1.25–4  Hz, *F*_1,55_ = 4.40, *p* = 0.04, *R*²_β*_ = 0.07, Fig. 3b), such that young-like frontal CEP was associated with increased, and presumably preserved SWE. We further found that SWE in the lower 0.5–1 Hz range was not significantly associated with GM volume (*F*_1,53_ = 1.56, *p* = 0.22), nor with whole-brain Aβ burden (*F*_1,53_ = 1.85, *p* = 0.18, Fig. 3c), contrasting with previous reports^11,27^, and was not linked to whole-brain tau burden (*F*_1,53_ = 0.05, *p* = 0.82). Likewise, CEP was not associated with any of the brain structural integrity measures (GM: *F*_1,53_ = 0.18, *p* = 0.67; Aβ: *F*_1,53_ = 0.16, *p* = 0.69; Tau: *F*_1,53_ = 0.48, *p* = 0.49). Frontal CEP relates therefore to a gold standard measure of sleep homeostasis known to decline in aging^13,15^, but is not significantly linked to the hallmarks of AD neuropathology.”
- In the “Discussion” section of page 4, the text:
  “Here, we confirm that brain activity during sleep is associated with Aβ burden also in healthy late middle-aged individuals (59.6 ± 5.5 years). Yet, in this relatively large (*N* = 60) younger sample, slow waves generation during sleep is not associated with tau burden nor with cognitive measures including memory, attention, and executive functions. We demonstrate instead that, at around 60 years, wake-dependent variations of basic cortical function is associated with cognitive fitness, independently of Aβ and tau burden and GM volume.”
  has been replaced with:
  “Here, we did not find a significant association between brain activity during sleep and Aβ burden in our sample of healthy late middle-aged individuals (59.6 ± 5.5 years). In addition, in this relatively large (*N* = 60) younger sample, slow waves generation during sleep is not associated with tau burden nor with cognitive measures, including memory, attention, and executive functions. We demonstrate instead that, at around 60 years, wake-dependent variations of basic cortical function are associated with cognitive fitness, independently of Aβ and tau burden and GM volume.”
- The figure legend for Fig. 3:
  “Cortical excitability, slow wave energy, and brain structural integrity. **a** Positive association between CEP and cumulated frontal NREM SWE in the lower range (0.75–1 Hz) during habitual sleep (*n* = 60; *F*_1,55_ = 5.35, *p* = 0.02, *R*²_β*_ = 0.09). **b** Positive association between CEP and cumulated frontal NREM SWE in the higher range (1.25–4 Hz) during habitual sleep (*n* = 60; *F*_1,55_ = 5.47, *p* = 0.02, *R*²_β*_ = 0.09). **c** Negative association between NREM SWE (0.75–1 Hz range) and whole-brain amyloid-beta burden (*n* = 60; *F*_1,53_ = 5.15, *p* = 0.03, *R*²_β*_ = 0.09). Simple regressions were used only for a visual display and do not substitute the GLMM outputs. Dotted lines represent 95% confidence interval of these simple regressions.”
  has been replaced with:
  “Fig. 3 Cortical excitability, slow wave energy, and brain structural integrity. **a** Positive association between CEP and cumulated frontal NREM SWE in the lower range (0.5–1 Hz) during habitual sleep (*n* = 60; *F*_1,55_ = 4.72, *p* = 0.03, *R*²_β*_ = 0.08). **b** Positive association between CEP and cumulated frontal NREM SWE in the higher range (1.25–4 Hz) during habitual sleep (*n* = 60; *F*_1,55_ = 4.40, *p* = 0.04, *R*²_β*_ = 0.07). **c** No significant association was found between NREM SWE (0.5–1 Hz range) and whole-brain amyloid-beta burden (*n* = 60; *F*_1,53_ = 1.85, *p* = 0.18). **c** is presented to allow comparison with the previous published version of the manuscript. Simple regressions were used only for a visual display and do not substitute the GLMM outputs. Dotted lines represent 95% confidence interval of these simple regressions.”

The errors have been corrected in the HTML and PDF versions of the Article.

