# Supplementary material for: Preserved wake-dependent cortical excitability dynamics predict cognitive fitness beyond age-related brain alterations
Source: Commun Biol. 2019 Dec 3;2:449. doi: 10.1038/s42003-019-0693-y (PMC6890637; doi:10.1038/s42003-019-0693-y)
Supplement: Supplementary file 2 — Description of Additional Supplementary Files [file 42003_2019_693_MOESM2_ESM.docx]

**Descriptions of additional supplementary files**

Supplementary_data_1 Excel file includes three data sheets containing raw values used to create figures 2, 3, and 4 in the manuscript.

The first sheet, named ‘Fig.2’, includes 4 columns:

- Column ‘subj’ refers to participants’ IDs attributed during recruitment.
- Column ‘phase’ corresponds to the 5 circadian phase related to the repeated TMS-EEG assessments, and were used to resample raw TMS-EEG data (0° = dim light melatonin onset time; 15° = 1h).
- Column ‘TEP_slope_resampled’ corresponds to the resampled cortical excitability values. See methods section for a detailed description of the computation of this value.
- Column ‘detrended_TEP_slope_resampled’ refers to the de-trended cortical excitability values.

Second sheet, named ‘Fig.3’, includes 5 columns:

- Column ‘subj’ refers to participants’ IDs attributed during recruitment.
- Column ‘CEP’ corresponds to the cortical excitability regulation profile across the 5 TMS-EEG assessments. See methods section for a detailed description of the computation of this value.
- Column ‘NREM_0.75-1Hz_energy’ corresponds to the accumulated power density in the 0.75-1Hz range during NREM sleep recorded during the baseline night under EEG. See methods section for a detailed description of the computation of this value.
- Column ‘NREM_1.25-4Hz_energy’ corresponds to the accumulated power density in the 1.25-4Hz range during NREM sleep recorded during the baseline night under EEG. See methods section for a detailed description of the computation of this value.
- Column ‘Whole-brain_Aß’ refers to whole-brain ^[18F]^Flutemetamol SUVR values assessed with amyloid-beta-PET.

Third sheet, named ‘Fig.4’, includes 6 columns:

- Column ‘subj’ refers to participants’ IDs attributed during recruitment.
- Column ‘CEP’ corresponds to the cortical excitability regulation profile across the 5 TMS-EEG assessments. See methods section for a detailed description of the computation of this value.
- Columns ‘Z_global_perf’, ‘Z_exec_perf’, ‘Z_mem_perf’, and ‘Z_att_perf’ correspond to the standardized cognitive performance scores for the global, executive, memory, and attentional functions, respectively.
