# Supplementary material for: Preserved wake-dependent cortical excitability dynamics predict cognitive fitness beyond age-related brain alterations
Source: Commun Biol. 2019 Dec 3;2:449. doi: 10.1038/s42003-019-0693-y (PMC6890637; doi:10.1038/s42003-019-0693-y)
Supplement: Supplementary file 4 — Reporting Summary [file 42003_2019_693_MOESM4_ESM.pdf]

nature research

Corresponding author(s): Gilles Vandewalle

Last updated by author(s): Oct 24, 2019

Reporting Summary

Nature Research wishes to improve the reproducibility of the work that we publish. This form provides structure for consistency and transparency in reporting. For further information on Nature Research policies, see Authors & Referees and the Editorial Policy Checklist.

Statistics

For all statistical analyses, confirm that the following items are present in the figure legend, table legend, main text, or Methods section.

n/a	Confirmed	
☐	☒	The exact sample size (*n*) for each experimental group/condition, given as a discrete number and unit of measurement
☐	☒	A statement on whether measurements were taken from distinct samples or whether the same sample was measured repeatedly
☐	☒	The statistical test(s) used AND whether they are one- or two-sided<br>*Only common tests should be described solely by name; describe more complex techniques in the Methods section.*
☐	☒	A description of all covariates tested
☐	☒	A description of any assumptions or corrections, such as tests of normality and adjustment for multiple comparisons
☐	☒	A full description of the statistical parameters including central tendency (e.g. means) or other basic estimates (e.g. regression coefficient) AND variation (e.g. standard deviation) or associated estimates of uncertainty (e.g. confidence intervals)
☐	☒	For null hypothesis testing, the test statistic (e.g. *F*, *t*, *r*) with confidence intervals, effect sizes, degrees of freedom and *P* value noted<br>*Give P values as exact values whenever suitable.*
☒	☐	For Bayesian analysis, information on the choice of priors and Markov chain Monte Carlo settings
☐	☒	For hierarchical and complex designs, identification of the appropriate level for tests and full reporting of outcomes
☐	☒	Estimates of effect sizes (e.g. Cohen's *d*, Pearson's *r*), indicating how they were calculated

Our web collection on statistics for biologists contains articles on many of the points above.

Software and code

Policy information about availability of computer code

Data collection	- TMS-EEG data were acquired using NBS and EximiaEEG softwares (Nexstim, Helsinki, Finland).<br>- Sleep data were acquired using RemLogic software (EMBLA, Natus Medical Incorporated, Planegg, Germany).<br>- Cognitive tests performed on computer were based on in-house codes implemented in MATLAB2013a (The Mathworks Inc., Natick, MA), except for the Mnemonic Similarity Task (MST, Stark et al., 2013).
Data analysis	- TMS-EEG data were preprocessed and analyzed with the SPM12 toolbox (https://www.fil.ion.ucl.ac.uk/spm/) implemented in MATLAB2013a.<br>- PET data were preprocessed and analyzed with the standard software ECAT v7.1 (Siemens/CTI, Knoxville, TN), the SPM12 toolbox and the PETPVC toolbox (Thomas et al., 2016).<br>- MRI data were preprocessed and analyzed with the SPM12 toolbox.<br>- Dim light melatonin onset data were analyzed with the Hockey-Stick software v1.5 (Danilenko et al., 2015).<br>- Actigraphy data were analyzed with Actiwatch Activity and Sleep Analysis software v7.43 (Cambridge Neurotechnology, UK).<br>- Sleep data were analyzed with MATLAB2013a and two validated algorithms for, respectively, automatic sleep scoring (ASEEGA, PHYSIP, Paris, France, Berthomier et al., 2007) and automatic artefact detection (Coppieters ’t Wallant et al., 2016).<br>- All statistical analyses were carried out in SAS 9.4 (SAS Institute, Cary, NC).<br>- Dependent variables distributions were assessed with 'allfitdist' function (developped by Mike Sheppard, https://nl.mathworks.com/matlabcentral/profile/authors/2201006-mike-sheppard) implemented in MATLAB2013a.

For manuscripts utilizing custom algorithms or software that are central to the research but not yet described in published literature, software must be made available to editors/reviewers. We strongly encourage code deposition in a community repository (e.g. GitHub). See the Nature Research guidelines for submitting code & software for further information.

Data

Policy information about availability of data

All manuscripts must include a data availability statement. This statement should provide the following information, where applicable:

- Accession codes, unique identifiers, or web links for publicly available datasets
- A list of figures that have associated raw data
- A description of any restrictions on data availability

The authors declare that the data supporting the findings of this study are available from the corresponding author upon request

Field-specific reporting

Please select the one below that is the best fit for your research. If you are not sure, read the appropriate sections before making your selection.

☒ Life sciences ☐ Behavioural & social sciences ☐ Ecological, evolutionary & environmental sciences

For a reference copy of the document with all sections, see nature.com/documents/nr-reporting-summary-flat.pdf

Life sciences study design

All studies must disclose on these points even when the disclosure is negative.

Sample size

Based on previous experiments (Ly et al. 2016; Gaggioni et al. 2019), we anticipated that the changes in cortical excitability across the 5 sessions of the protocol would be of small to medium effect size. We therefore aimed at being able to detect effect size r > 0.15. According to G*Power software (Faul et al., 2007), in the linear model including repeated measures, with power set at beta = 0.8 and significance threshold set at alpha = 0.05, this corresponds to a sample size of N = 55. With our sample of 60 subjects, we should be able to detect effect sizes > 0.14.

Data exclusions

One participant was excluded from the sample for all analyses because of outlier values on both PET assessments (> 6 standard deviations from the mean)

Replication

Our findings are new and based on a unique sample and a unique approach. We cannot therefore reproduce any of our findings in independent samples. Yet, we replicate previous findings from the literature:
- Association between slow oscillations and amyloid-beta burden (Mander et al. 2015);
- Wake-dependent dynamics in cortical excitability (Huber et al. 2013; Ly et al. 2016; Gaggioni et al. 2019);
- Association between cortical excitability and cognition (Ly et al. 2016; Gaggioni et al. 2019)

Randomization

Randomization was not relevant in our study as we did not have group allocation, neither during data collection nor data analysis. We used 'subject' as a random effect in all our statistical model to account for potential sampling bias.

Blinding

Blinding was not relevant in our study as we did not have group allocation, neither during data collection nor data analysis

Reporting for specific materials, systems and methods

We require information from authors about some types of materials, experimental systems and methods used in many studies. Here, indicate whether each material, system or method listed is relevant to your study. If you are not sure if a list item applies to your research, read the appropriate section before selecting a response.

Materials & experimental systems

n/a	Involved in the study	
☒	☐	Antibodies
☒	☐	Eukaryotic cell lines
☒	☐	Palaeontology
☒	☐	Animals and other organisms
☐	☒	Human research participants
☒	☐	Clinical data

Methods

n/a	Involved in the study	
☒	☐	ChIP-seq
☒	☐	Flow cytometry
☐	☒	MRI-based neuroimaging

Human research participants

Policy information about studies involving human research participants

Population characteristics

Healthy older individuals aged 50-70 years were recruited. Exclusion criteria were: clinical symptoms of cognitive impairment (Dementia rating scale < 130; Mini mental state examination < 27); Body Mass Index (BMI) ≤ 18 and ≥ 29; recent psychiatric history or severe brain trauma; addiction, chronic medication affecting the central nervous system; smoking, excessive alcohol (>

	14 units/week) or caffeine (> 5 cups/day) consumption; shift work in the past 6 months; transmeridian travel in the past two months; anxiety, as measured by the 21-item self-rated Beck Anxiety Inventory (BAI ≥ 10); depression, as assessed by the 21-item self-rated Beck Depression Inventory (BDI ≥ 14). Participants with sleep apnea (apnea-hypopnea index ≥ 15/hour) were excluded based on an in-lab screening night of polysomnography.
Recruitment	Participants were recruited via advertisements in local newspapers, local broadcasts channels, university website, and word of mouth.
Ethics oversight	This research was approved by the Ethics Committee of the Faculty of Medicine at the University of Liège, Liège, Belgium

Note that full information on the approval of the study protocol must also be provided in the manuscript.

Magnetic resonance imaging

Experimental design

Design type	We performed structural acquisitions that were used for TMS neuronavigation and that aimed at assessing brain integrity markers of whole-brain and regional grey matter volumes. Moreover, MRI structural data were used for anatomical reference in spatial normalization of PET images.
Design specifications	Acquisition time of about 5 min (T1 MPRAGE) + 19 min (MPM protocol) + 4 min (B1 mapping).
Behavioral performance measures	No behavioral performance was measured during MRI acquisition.

Acquisition

Imaging type(s)	Structural T1 acquisition for TMS neuronavigation and structural multiparametric maps (MPM) acquisition for grey matter volume extraction and anatomical reference in spatial normalization of PET images.
Field strength	3T
Sequence & imaging parameters	Structural T1 for TMS neuronavigation: standard 3D T1-weighted image MPRAGE (TR/TE/TI = 1900/2.19/900ms, FA = 9°, FoV 256 x 240 mm²; 1 x 1 x 1 mm³; matrix size = 256x240x224; acceleration factor in phase-encoding direction R=2) MPM sequences: three co-localized 3D multi-echo fast low angle shot (FLASH) acquisitions with predominantly proton density weighting (PDw, TR/FA=23.7 ms/6°), T1 weighting (T1w: 18.7 ms/20°), and magnetization transfer weighting (MTw: 23.7 ms/6°; excitation preceded by an off-resonance Gaussian MT pulse of 5 ms duration, 220° nominal flip angle, 2 kHz frequency offset), with a resolution of 1 x 1 x 1 mm³. Two additional calibration sequences to correct for inhomogeneities in the RF transmit field.
Area of acquisition	Whole brain

Diffusion MRI ☐ Used ☒ Not used

Preprocessing

Preprocessing software	MPM data were preprocessed using SPM12 implemented in MATLAB2013a. MTw volumes were segmented to estimate whole-brain and regional (cf. anatomical locations below) grey matter volume for each individual.
Normalization	MRI data were spatially normalized into a study-specific template with the Diffeomorphic Anatomical Registration Through Exponentiated Algebra (DARTEL) toolbox in SPM12.
Normalization template	The normalization template was a DARTEL study-specific template.
Noise and artifact removal	Not applicable to the structural acquisitions described here.
Volume censoring	Not applicable to the structural acquisitions described here.

Statistical modeling & inference

Model type and settings	We extracted whole-brain and ROI grey matter volume which we used as covariates in statistical models including brain integrity markers. No statistical tests were performed on brain structural maps per se.
Effect(s) tested	We tested whether the inclusion of MRI-derived index of grey matter integrity modulated the observed relationship between cognitive performance and TMS-EEG-derived values.

Specify type of analysis: ☐ Whole brain ☐ ROI-based ☒ Both

Anatomical location(s)	Anatomical locations for ROI-based grey matter integrity values were extracted using the Anatomical Labeling Atlas (AAL2, Tzourio-Mazoyer 2002). ROI were defined based on the classical brain networks underlying the investigated cognitive functions of memory, attention, and executive abilites (Niendam, 2012; Collette et al., 2002; Rugg & Vilberg, 2013; Jeong et al., 2015; Fan et al., 2005; Corbetta & Shulman, 2002).

Statistic type for inference (See Eklund et al. 2016)	No statistical tests were performed on brain structural maps per se.
Correction	No statistical tests were performed on brain structural maps per se.

Models & analysis

n/a	Involved in the study
☒	☐ Functional and/or effective connectivity
☒	☐ Graph analysis
☒	☐ Multivariate modeling or predictive analysis
